# Recovery of the Cell Cycle Inhibition in CCl_4_-Induced Cirrhosis by the Adenosine Derivative IFC-305

**DOI:** 10.1155/2012/212530

**Published:** 2012-09-27

**Authors:** Victoria Chagoya de Sánchez, Lidia Martínez-Pérez, Rolando Hernández-Muñoz, Gabriela Velasco-Loyden

**Affiliations:** Departmento de Biología Celular y Desarrollo, Instituto de Fisiología Celular, Universidad Nacional Autónoma de México, 04510 México, DF, Mexico

## Abstract

*Introduction*. Cirrhosis is a chronic degenerative illness characterized by changes in normal liver architecture, failure of hepatic function, and impairment of proliferative activity. The aim of this study is to know how IFC-305 compound induces proliferation of the liver during reversion of cirrhosis. *Methods*. Once cirrhosis has been installed by CCl_4_ treatment for 10 weeks in male Wistar rats, they were divided into four groups: two received saline and two received the compound; all were euthanized at 5 and 10 weeks of treatment. Liver homogenate, mitochondria, and nucleus were used to measure cyclins, CDKs, and cell cycle regulatory proteins PCNA, pRb, p53, E2F, p21, p27, HGF, liver ATP, and mitochondrial function. *Results*. Diminution and small changes were observed in the studied proteins in the cirrhotic animals without treatment. The IFC-305-treated rats showed a clear increase in most of the proteins studied mainly in PCNA and CDK6, and a marked increased in ATP and mitochondrial function. *Discussion/Conclusion*. IFC-305 induces a recovery of the cell cycle inhibition promoting recovery of DNA damage through the action of PCNA and p53. The increase in energy and preservation of mitochondrial function contribute to recovering the proliferative function.

## 1. Introduction

Cirrhosis is one of the most common causes of mortality worldwide and is induced by chronic liver injury, such as that produced by alcoholic hepatitis, viral hepatitis, autoimmune disease against biliary ducts, and metabolic diseases such as hepatic steatosis.

Cirrhosis is a complex process for which no effective treatment has been developed [1]. It is accompanied by a change in the architecture of the liver with loss of function and it is considered irreversible, mainly due to the increased deposition of connective tissue resulting from an increased collagen synthesis accompanied by a deficient degradation of deposited collagen [2]. Another important factor is the well-known impaired capability of the liver to regenerate after hepatic resection [3]. The severity of liver fibrosis is considered to be related with impaired regenerative capacity, suggesting the arrest of cell cycle progression [4]. Cirrhosis development is preceded by inflammation, apoptosis, and fibrosis processes that are accompanied by energetic unbalance as well as oxidative damage induced by reactive oxygen species that could result in chromosomal instability which induces injury in the check points of the cellular cycle causing an impaired regenerative capacity. Our research group has been studying the hepatoprotective effect of adenosine and an adenosine derivative, IFC-305, in the development and in the reversion of established cirrhosis on a rat model of liver cirrhosis induced by CCl_4_ [5–7]. Adenosine is a purine nucleoside considered an autocoid, present within and outside the cells that acts as chemical messenger with autocrine, paracrine and endocrine actions. It is mostly formed by *the novo* purine synthesis principally in the liver [8], by phosphohydrolysis of adenine nucleotides via endo- and ecto-5′nucleotidases or by hydrolysis of S-adenosyl homocysteine in the methylation pathway [9]. Extracellular adenosine can exert its function through activation of adenosine receptors (A1, A2a, A2b, and A3) or can be transported into the cells by the nucleoside transporters (ETN1). Within the cell adenosine can be phosphorylated by adenosine kinase, deaminated to inosine by adenosine deaminase, or transformed in S-adenosyl-homocysteine by S-adenosyl-homocysteine hydrolase [9]. Its metabolism is very active resulting in a short half-life of the nucleoside but with an action as metabolic modulator. The derivative IFC-305 has a longer half-life in the liver potentiating the beneficial effects of adenosine on CCl_4_-induced cirrhosis in rats [10]. Adenosine presents interesting effects in hepatic metabolism: it increases the energy charge of the hepatocyte, augments the synthesis of hepatic glycogen, and inhibits fatty acid oxidation [11–13]; it also modulates the redox state of the cell through maintaining the structure and function of the mitochondria [14]. In acute hepatotoxicity, induced by ethanol or CCl_4_, adenosine prevents the fatty liver induced by the toxics, restitutes the energetic balance, the redox equilibrium, mitochondrial function, and prevents oxidative stress by diminishing the reactive oxygen species and increasing antioxidant defenses [5, 15–18]. In preestablished cirrhosis, adenosine increases collagen degradation, prevents its accumulation, preserves the energy and functional states of the liver, increases DNA synthesis, the mitotic index, and the expression of proliferating cell nuclear antigen (PCNA) [7]. Recently, we have demonstrated that adenosine administration accelerates progression of the cell cycle during liver regeneration in rats subjected to one-third hepatectomy [19]. Some of these effects, such as prevention of collagen accumulation and increase in DNA synthesis, have been reproduced with the IFC-305 [5, 10]. Moreover, microarray studies showed that 414 genes modified in cirrhosis are decreased to 263 with IFC-305 treatment and the downregulated or upregulated genes showed a tendency to normalize with the compound treatment, among them there are 24 genes involved in the cell cycle [10]. On the other hand, it has been shown that the cell cycle-related molecules play essential roles in hepatocyte proliferation. Specifically, G1-related molecules are important because they are a requisite to enter into the cell cycle from the quiescent state. Although the role of these molecules of the cell cycle has not been studied in detail during cirrhosis development, we hypothesize that the action of adenosine and its derivative IFC-305 must be related with the molecules and regulation points of the cell cycle. Sweet and Singh [20] showed that the progression through the cell cycle is sensitive to changes in mitochondrial-derived ATP and describes two energetic checkpoints at the G_1_-S and the G_2_-M as borders that prevent the progression of the proliferation cycle.

Numerous studies employing inhibitors of the mitochondrial function have demonstrated that cell division is sensitive to alterations of the energy pool [21–23]. In order to know how the IFC-305 repairs the proliferative function in the cirrhotic liver, we studied the role of cyclins of the cellular cycle (cyclins D, E, A, and B), cyclin-dependent kinase (CDKs), some other proteins such as PCNA, pRb, E2F1, p53, and the hepatic growth factor (HGF) and correlated them with some parameters of the mitochondrial function in experimental cirrhosis induced by CCl_4_.

## 2. Materials and Methods

### 2.1. Animal's Treatment and Induction of Cirrhosis by CCl_4_

 Male Wistar rats (*n* = 30) weighing 100 to 110 g were rendered cirrhotic by chronic treatment with CCl_4_. Animals were intraperitoneally injected (0.4 g/kg) three times a week during 10 weeks with a solution 1/6 of CCl_4_ in vegetable oil. Cirrhosis-induced rats were divided into five groups (six animals per group). At time zero (T0 group), rats were euthanized 24 hours after cessation of CCl_4_, two groups were treated with saline solution during 5 (SS5) or 10 weeks (SS10) and two groups were treated with IFC305 at a 50 mg/kg dose, three times weekly, for 5 (IFC5) or 10 weeks (IFC10), all the experiments include rats without treatment (C). Animals were euthanized with a sodium pentobarbital overdose (63 mg/kg animal weight); the liver was recovered, rinsed in saline solution, and frozen with liquid nitrogen. Animals were obtained and housed from the animal facility of the National Autonomous University of Mexico (UNAM). All procedures were conducted according to our institutional guidelines for the care and use of laboratory animals.

### 2.2. Plasma Collection

Animals were anesthetized as indicated and about 10 mL of whole blood was collected by cardiac puncture into syringes filled with 1ml of 0.25 M EDTA, pH 8.0. Cells were removed from plasma by centrifugation for 10 minutes at 3000 rpm at 4°C. Following centrifugation, the supernatant (plasma) was stored into 0.5 mL aliquots at −70°C until use. 

### 2.3. Liver Subcellular Fractionation

Liver samples were homogenized (1 : 10 w/v) in a medium containing 250 mmol/L sucrose, 10 mmol/L Trizma base, and 1 mmol/L EDTA. The homogenate was centrifuged at 3500 rpm for 5 min at 4°C, the supernatant was used as total liver protein extract, or the supernatant was centrifuged at 8500 rpm for 10 min at 4°C to obtain the mitochondrial pellet. Mitochondrial respiration and phosphorylation were recorded polarographically with a Clark-type oxygen electrode in 3 mL of a medium containing 225 mM sucrose, 10 mM KCl, 5 mM MgC1_2_, 10 mM potassium phosphate, 10 mM Tris-HCl, and 0.05% fatty-acid-free albumin (pH 7.4). Glutamate and malate (10 and 1 mM, resp.) were used as substrates for site I. Mitochondrial state 3 was initiated by addition of ADP (266 *μ*mol/liter final concentration). The membrane potential (ΔΨ) was measured by monitoring the movements of tetraphenyl phosphonium (TPP+)^2^ across the mitochondrial membrane as previously described [24]. Protein concentration was determined by the Lowry method [25]. 

### 2.4. Liver Nuclear Protein Extractions

The nuclear fraction was obtained by the method used by Sindić et al. [26]. In brief, livers were homogenized in ice-cold 10 mM HEPES buffer, pH 7.5, containing 5 mM MgCl_2_, 25 mM KCl, and the protease inhibitor cocktail. Homogenates were spun through a discontinuous sucrose gradient and resuspended in the indicated medium [26]. Protein quantification was determined with the method of Bradford [27].

### 2.5. ATP Determination

For adenine nucleotide ATP determination, 300 mg of the liver was extracted with 8% perchloric acid, after centrifugation the sample was neutralized with 4 M K_2_CO_3_. ATP was quantified by reversed- phase high-performance liquid chromatography [28]. 

### 2.6. Western Blot Analysis

Liver nuclear protein or total liver extract (30 *μ*g of protein/well) was electrophoresed in SDS-polyacrylamide gels and transferred to nitrocellulose membranes. Membranes were blocked for 2 h with 5% nonfat dry milk in TBST (50 mM Tris, 150 mM NaCl at pH 7.4, and 0.05% Tween 20). The blots were incubated overnight at 4°C with primary antibodies against PCNA (Upstate, Lake Placid, NY, USA), adenosine Receptors A1, A2a, A2b, A3 (Alpha Diagnostics Intl, San Antonio, TX, USA), cyclin D1, cyclin E, cyclin A, cyclin B1, CDK4, CDK6, p21, p27, phospho-Rb (Ser 795), E2F1, DP1, p53, HGF*α*, *β*-actin (Santa Cruz Biotechnology, Santa Cruz, CA), MDM2, and c-Met (Millipore, Temecula, CA, USA). Primary antibody binding was detected with the respective horseradish peroxidase-conjugated secondary antibody. Protein bands were visualized by using chemiluminescence luminol reagent (Santa Cruz Biotechnology). Densitometric analyses of bands were done with the Quantity One software (Bio-Rad, Hercules, CA, USA).

### 2.7. RNA Isolation and Quantitative RT-PCR Analysis

Frozen liver samples were used for total RNA isolation by Tripure-based extraction (Roche Applied Science). Quantity and purity were determined by measuring the optical density at 260/280 nm in a UV-spectrophotometer. RNA quality was verified by agarose-gel electrophoresis and rRNA 28S/18S > 1.7 ratios were used. cDNA synthesis was performed from 2 *μ*g of total RNA using the high-capacity cDNA Archive Kit (Applied Biosystems, Foster City, CA, USA) following the manufacturer's protocol. PCR reactions were optimized for all genes to obtain one PCR product that corresponded to the size predicted by the primer design. Real-time quantitative PCR reactions (qPCR) were done in the ABI PRISM 7000 Sequence Detection System (Applied Biosystems) using Platinum SYBR Green qPCR Super Mix-UDG with ROX Kit (Invitrogen, Carlsbad, CA USA) according to the manufacturer's instructions. All quantitative PCR assays were performed independently in at least three animals/group in triplicate with the corresponding standard dilution curves. Relative mRNA transcript levels were expressed in arbitrary units as *n*-folds of untreated control after normalization to the acidic ribosomal protein (Arbp) mRNA. The primers used were cyclin D1 forward 5^'^-GCAAGAATGTGCCAGACTCA-3^'^ and reverse 5^'^-ACGGAGATGTGGTCTCCTTG-3^'^; Rb forward 5^'^-CACGAAAAAGCAACCCTGAT-3^'^ and reverse 5^'^-TCTGATGGCTGATCACTTGC-3^'^; p53 forward 5^'^-GCTTCGAGATGTTCCGAGAG-3^'^ and reverse 5^'^-CTTCGGGTAGCTGGAGTGAG-3^'^; Arbp, forward 5^'^-AGGTGGTGCTGATGGGCA-3^'^ and reverse 5^'^-CCTCCGGATGTGAGGCAG-3^'^.

### 2.8. PCNA Immunohistochemistry

Sections of liver were fixed in 10% neutral buffered formalin and embedded in paraffin. For PCNA immunohistochemistry, liver sections of 4 *μ*m thick were mounted in lysine-coated slides, deparaffinized in xylene, and passed through graded alcohols. The Dako EnVision+ System-HRP (DAB) (Dako, Carpinteria, CA, USA) was used. Previously, slides were immersed in antigen-retrieval solution using Citrate Buffer (10 mM Citric Acid, 0.05% Tween 20, pH 6.0) in a plastic Coplin jar placed in a microwave oven set on middle power for 10 min. After cooling at room temperature, sections were incubated for 10 min with the peroxidase block, and then incubated 10 min with Protein Block, Serum-Free (DakoCytomation, Denmark). Slides were incubated overnight at 4°C with the primary antibody, monoclonal anti-PCNA clone PC10 (Dako, Carpinteria, CA, USA) at a dilution of 1 : 100. The next day, slides were incubated for 60 min with the labeled polymer-HRP anti-mouse at room temperature. Tissue sections were then incubated with DAB substrate buffer and counterstained with hematoxylin and coverslipped with Entellan (Merck, Darmstadt, Germany). PCNA-positive hepatocytes were counted from images captured with Evolution/QImaging Digital Camera (Media Cybernetics, Bethesda, MD, USA) of five randomly chosen fields by light microscope using a 40x objective magnification from *n* = 3 rats. Counting of PCNA-positive hepatocytes was done with the Image-Pro Plus 7 (Media Cybernetics, Inc., Bethesda, MD, USA). The hepatocytes were considered positive for PCNA when the immunostaining was present in the nuclei or cytoplasm, cells in S + G1 + G2 + M as it has been described by others [29, 30].

### 2.9. Statistical Analysis

 All values were expressed as mean ± SEM of three independent experiments. Statistical analysis was performed using the unpaired, nonparametric Student's *t*-test. Differences with a *P* value of less than 0.05 were considered statistically significant.

## 3. Results

### 3.1. Proliferating Cell Nuclear Antigen (PCNA) in the Cirrhotic Rats Treated with IFC-305

**Figure 1:**
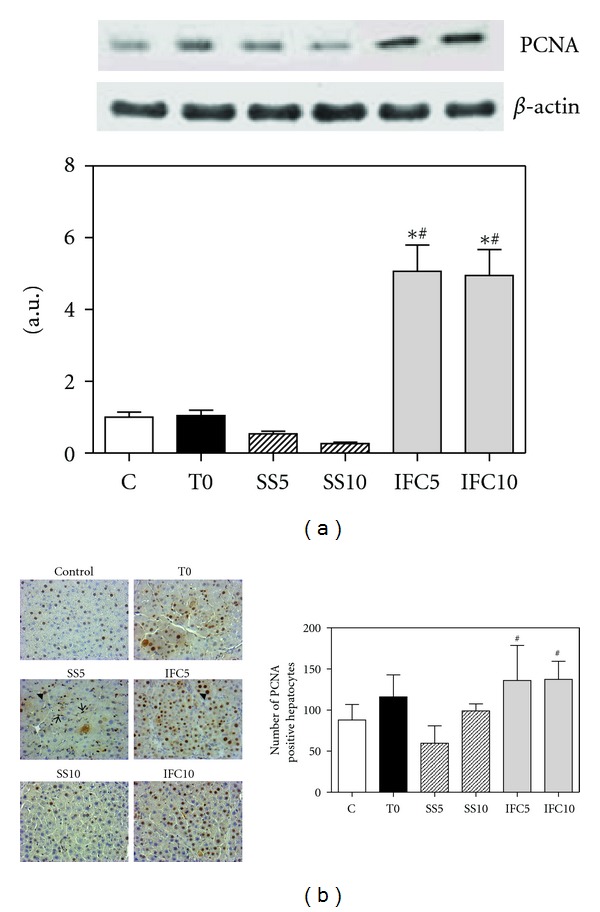
Effect of IFC305 treatment on the liver PCNA protein expression in CCl_4_-induced cirrhosis in rats. (a) Protein expression of PCNA and the house keeping gen *β*-actin from liver nuclear extracts was determined by western blot analysis. A representative western blot image is shown. The bar graph represents the densitometry analysis expressed as arbitrary units of the mean ± SEM from 3 rats/group; values were normalized to *β*-actin immunodetection. (b) Immunohistochemical analysis of PCNA expression. Hepatocyte (arrowhead) and nonparenchymal cell (arrow) are marked. The number of PCNA positive hepatocytes was quantified as described in Section 2. *Statistical difference (*P* < 0.01) when compared to C group. ^#^Statistical difference (*P* < 0.01) when compared to their respective experimental group SS5 or SS10.

PCNA is an auxiliary protein of the DNA polymerase delta and is an excellent marker of cell proliferation present at the beginning of the S phase. Nuclear cell expression of the protein is presented in Figure 1(a), showing a diminution at 5 and 10 weeks after established cirrhosis in the presence of saline, whereas in the presence of IFC-305, an 8- to 10-fold increase was noticed, supporting the effect of the compound on hepatic proliferation. We also analyzed proliferating cells by immunohistochemical identification of PCNA in liver tissue sections. Although the difference was less pronounced than in the western blot analysis, we also observed an increase in PCNA expression in hepatocytes of liver rats treated with the compound (Figure 1(b)). We observed that in the group of rats at 5 weeks of cirrhosis progress, there was an increase in proliferation of other cells than hepatocytes, nonparenchymal cells, such as hepatic stellate cells, Kupffer or endothelial cells, although the identity of them should be confirmed (Figure 1(b)). 

### 3.2. Cyclins D1, E, A, and B1

**Figure 2:**
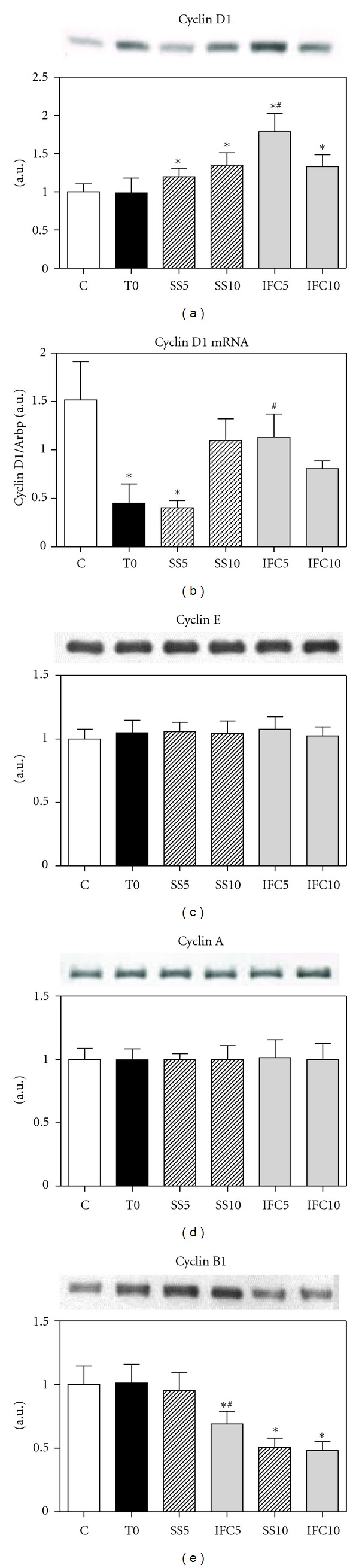
Effect of IFC305 treatment on cyclin D1, cyclin E, cyclin A, and cyclin B1 protein expression in CCl_4_-induced cirrhosis in rats. (a), (c), (d), and (e) Expression of the indicated proteins from liver nuclear extracts was determined by western blot analysis. A representative western blot image of each one is shown. The bar graph represents the densitometry analysis expressed as arbitrary units of the mean ± SEM from 3 rats/group, values were normalized to *β*-actin immunodetection (image, Figure 1(a)). *Statistical difference (*P* < 0.05) when compared to C group. ^#^Statistical difference (*P* < 0.05) when compared to their respective experimental group SS5 or SS10. (b) Effect of IFC305 treatment on cyclin D1 mRNA expression in CCl_4_-induced cirrhosis in rats. RNA was isolated from liver and mRNA expression for cyclin D1 was analyzed by quantitative RT-PCR as described in Section 2. Arbitrary units were normalized with Arbp mRNA gene expression level. Data represent mean ± SEM from 3 rats/group. *Statistical difference (*P* < 0.05) compared to C group. ^#^Statistical difference (*P* < 0.05) when compared to their respective experimental group SS5 or SS10.

Cyclins D and E belong to the G1 phase and are fundamental to initiate the cell cycle, whereas cyclins A and B are mitotic cyclins for the G2 M phase. These molecules are induced by mitogenic signals and extracellular growth factors, they have a short half-life and they are ubiquitinated and destroyed by the proteosome. The level of cyclin D in cirrhotic animals at T0 is similar to that of the control rats, but, at 10 weeks of progress, it increased 35%; animals treated with IFC-305 for 5 weeks showed a 77% increase, and a similar value at 10 weeks of treatment to that observed in nontreated rats (Figure 2(a)), these results are supported by the increase in mRNA of cyclin D1 (Figure 2(b)). Cyclins E and A did not show noticeable changes in the cirrhotic rats treated or not treated with the compound (Figures 2(c) and 2(d)). Cyclin B1 decreased by 30 and 52% at 5 and 10 weeks of IFC-305, respectively, in relation to the cirrhotic animals at T0 and after 10 weeks of progress, the cirrhotic rats treated with saline also decreased cyclin B1 in a similar way to that observed in the rats treated with the compound (Figure 2(e)). It is interesting to keep in mind that the degradation of cyclin B1 is important for metaphase-anaphase transition and progression of the cell cycle [31].

### 3.3. Cyclin Dependent Kinases (CDK4, CDK6) and Cell Cycle Inhibitors (p21, p27)

**Figure 3:**
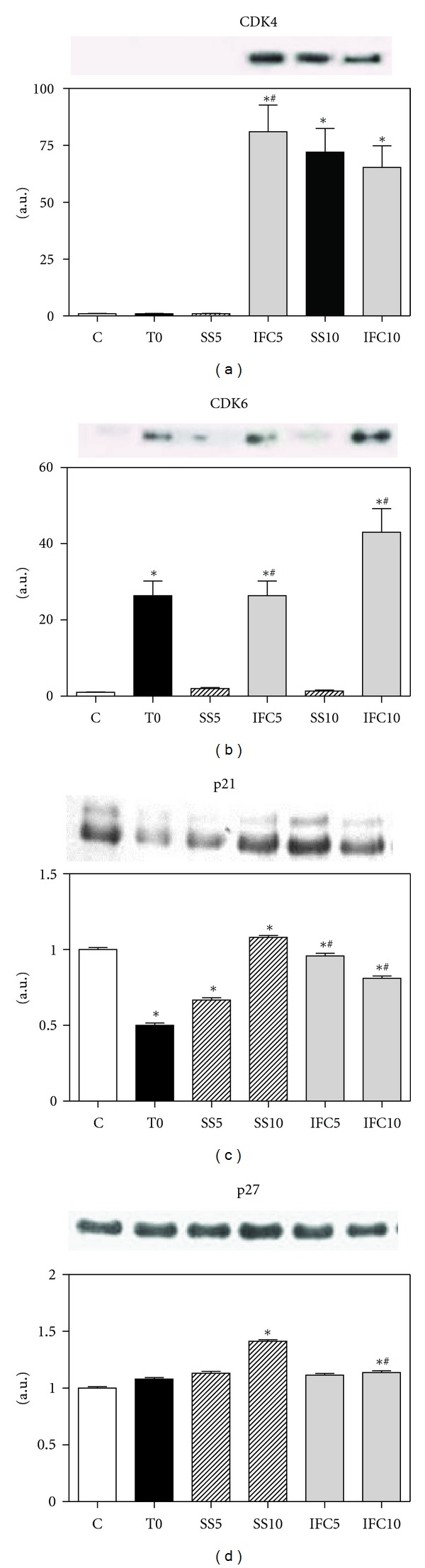
Effect of IFC305 treatment on CDK4, CDK6, p21, and p27 proteins expression in cirrhotic livers. (a), (b), (c), and (d) Expression of the indicated proteins from liver nuclear extracts was done by western blot analysis. A representative western blot image is shown. The bar graph represents the densitometry analysis expressed as arbitrary units of the mean ± SEM from 3 rats/group; values were normalized to *β*-actin immunodetection (image, Figure 1(a)). *Statistical difference (*P* < 0.05) when compared to C group. ^#^Statistical difference (*P* < 0.05) when compared to their respective experimental group SS5 or SS10.

For the progression of the cell cycle to S phase, cyclin D1 associates with a cyclin dependent kinase 4 or 6 (CDK4/CDK6) to form an active complex, cyclin-D/CDK4/CDK6 (Figures 3(a) and 3(b)). CDK4 increased at 5 and 10 weeks of IFC-305 treatment but also at 10 weeks of progress without treatment. CDK6 was present in the cirrhotic group at T0 and was maintained at 5 weeks of IFC-305 treatment with an important increase at 10 weeks, a marked decrease was seen in the cirrhotic rats without treatment. Regarding expression of p21 and p27 cell cycle inhibitors (Figures 3(c) and 3(d)), p21 showed a 40% decrease in the cirrhotic animals at T0, recuperating its value after 5 or 10 weeks of progress with and without treatment; p27 did not diminish in cirrhosis, but a 30 and 15% increase was noticed after 10 weeks of progress with and without treatment, respectively. 

### 3.4. Expression of Phospho-Rb and the Transcription Factor E2F-1

**Figure 4:**
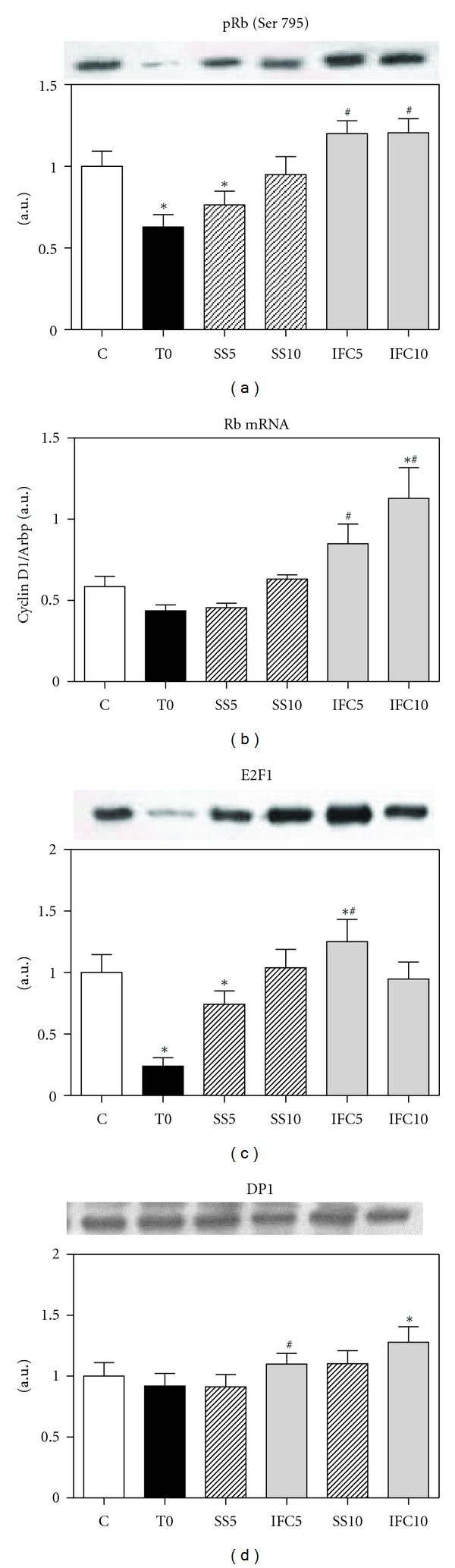
Effect of IFC305 treatment on phospho-Rb (Ser 795), E2F1, and DP1 proteins expression in cirrhotic livers. (a), (c), and (d) Expression of the indicated proteins from liver nuclear extracts was done by western blot analysis. A representative western blot image of each one is shown. The bar graph represents the densitometry analysis expressed as arbitrary units of the mean ± SEM from 3 rats/group; values were normalized to *β*-actin immunodetection (image, Figure 1(a)). *Statistical difference (*P* < 0.05) when compared to C group. ^#^Statistical difference (*P* < 0.05) when compared to their respective experimental group SS5 or SS10. (b) Effect of IFC305 treatment on Rb mRNA expression in CCl_4_-induced cirrhosis in rats. RNA was isolated from liver and mRNA expression for Rb was analyzed by quantitative RT-PCR as described in Section 2. Arbitrary units were normalized with Arbp mRNA gene expression level. Data represent mean ± SEM from 3 rats/group. *Statistical difference (*P* < 0.05) compared to C group. ^#^Statistical difference (*P* < 0.05) when compared to their respective experimental group SS5 or SS10.

An important function of the complex D/CDK4/6 is the phosphorylation of protein Rb, releasing the transcriptional factor E2F1 that activates the DNA synthesis genes and induces the entry of the cell into the S phase. A small increase in the phospho-Rb was observed in the group treated with the compound, but a significant increase in its mRNA at 5 and 10 weeks of IFC-305 treatment was observed. A 70% increase was observed in E2F1 that form a functional heterodimer with DP1 [32] in the nucleus obtained from cirrhotic rats treated for 5 weeks with IFC-305. The expression of DP1 also increased with the compound suggesting that the complex E2F/DP1 induces the S phase (Figures 4(a), 4(b), 4(c), and 4(d)).

### 3.5. Expression of p53 and the Protein MDM2

**Figure 5:**
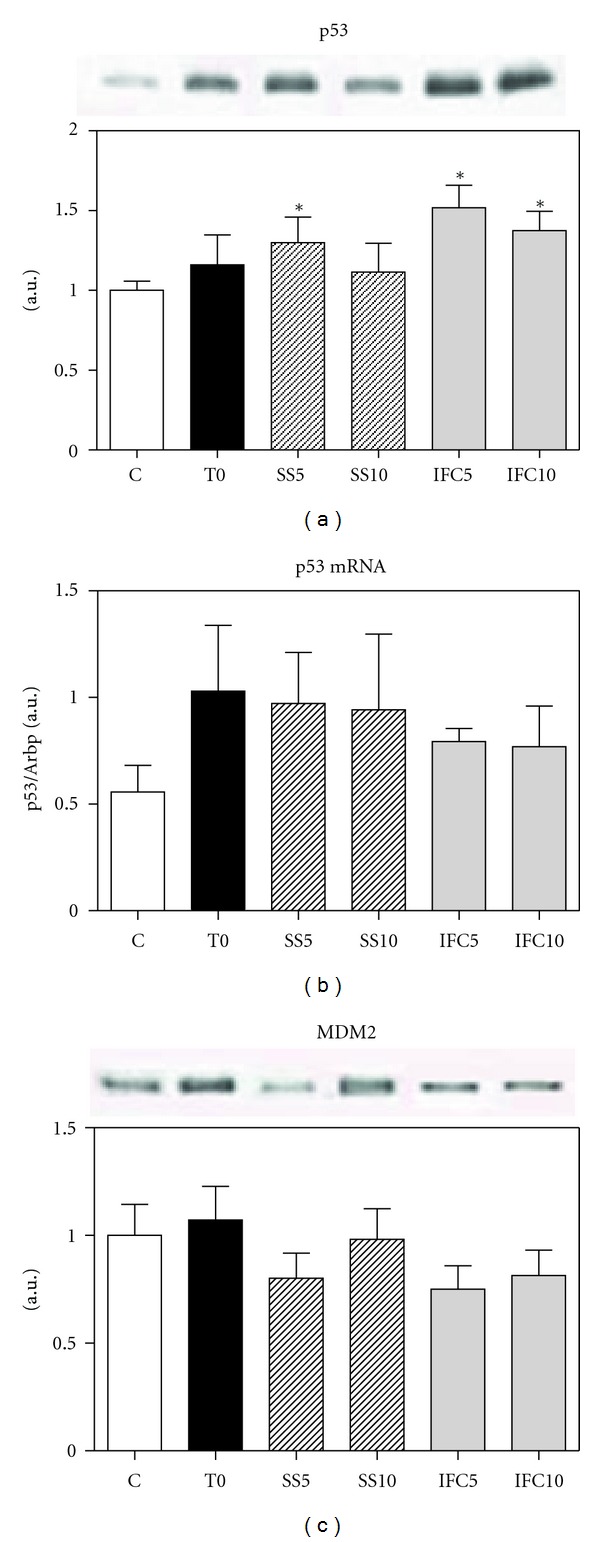
Effect of IFC305 treatment on p53 and MDM2 proteins expression in cirrhotic livers. (a) and (c) Expression of the indicated proteins from liver nuclear extracts was done by western blot analysis. A representative western blot image of each one is shown. The bar graph represents the densitometry analysis expressed as arbitrary units of the mean ± SEM from 3 rats/group; values were normalized to *β*-actin immunodetection (image, Figure 1(a)). *Statistical difference (*P* < 0.05) when compared to C group. ^#^Statistical difference (*P* < 0.05) when compared to their respective experimental group SS5 or SS10. (b) Effect of IFC305 treatment on p53 mRNA expression in CCl_4_-induced cirrhosis in rats. RNA was isolated from liver and mRNA expression for Rb was analyzed by quantitative RT-PCR as described in Section 2. Arbitrary units were normalized with Arbp mRNA gene expression level. Data represent mean ± SEM from 3 rats/group. *Statistical difference (*P* < 0.05) when compared to C group. ^#^Statistical difference (*P* < 0.05) when compared to their respective experimental group SS5 or SS10.

p53 has two important functions in the cell cycle, when there is DNA damage it induces arrest of the cycle at the check points of G1/S or G2/M. On the other hand, it induces the DNA repairing enzymes. Once DNA is repaired, it stimulates MDM2 synthesis for p53 degradation in the proteosome. In the cirrhotic T0 animals, there was a 19% increase in p53 expression, which was also observed at 5 and 10 weeks versus control rats; in cirrhotic animals treated with the compound, the increase was of 55 and 40% at 5 and 10 weeks of treatment, respectively (Figure 5(a)). When the gene expression was measured, only the cirrhotic groups presented an increase (Figure 5(b)), suggesting that IFC-305 did not increase the mRNA and that the observed increase in p53 protein could be due to a diminution in its degradation, as observed in the 25% decrease of protein expression of MDM2 in the IFC-305-treated rats indicating a diminution in p53 degradation (Figure 5(c)).

### 3.6. Hepatocyte Growth Factor/c-Met

**Figure 6:**
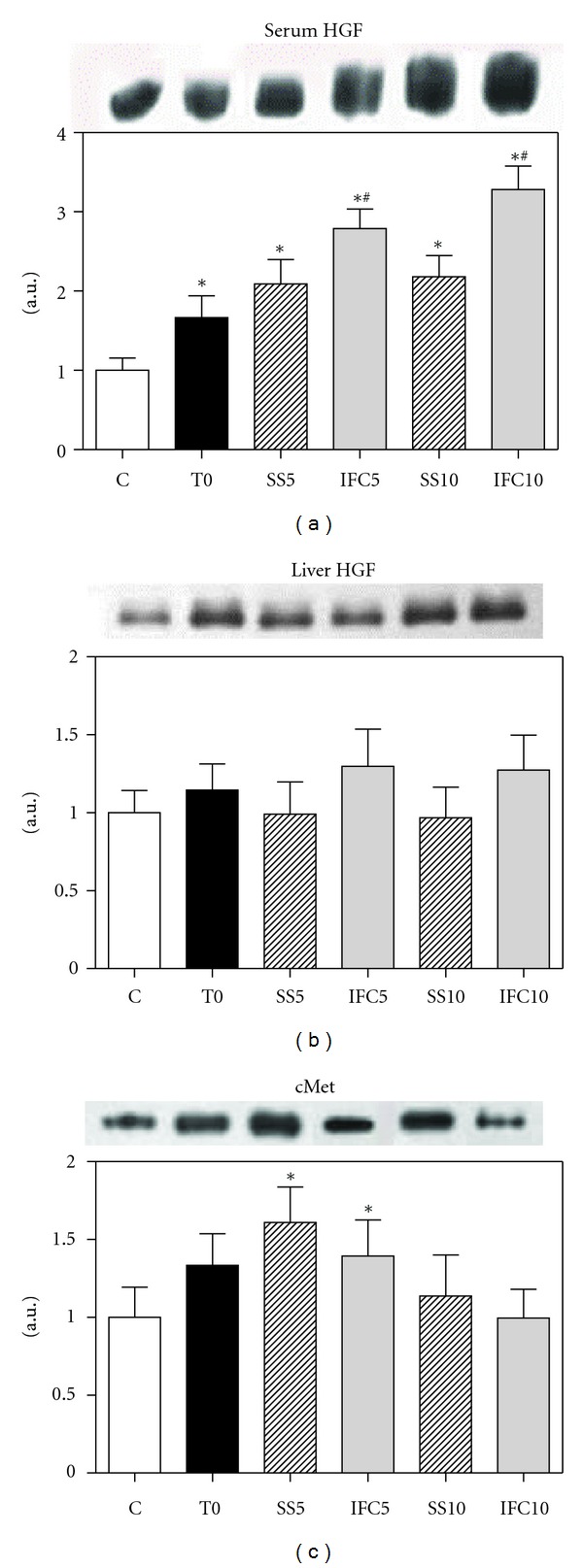
Effect of IFC305 treatment on serum HGF, liver HGF, and cMet proteins expression in CCl_4_-induced cirrhosis in rats. (a), (b), and (c) Expression of the indicated proteins from serum or liver extracts was done by western blot analysis. A representative western blot image of each one is shown. The bar graph represents the densitometry analysis expressed as arbitrary units of the mean ± SEM from 3 rats/group; values were normalized to *β*-actin immunodetection (image, Figure 1(a)). Values from serum (a) were not normalized. *Statistical difference (*P* < 0.05) when compared to C group. ^#^Statistical difference (*P* < 0.05) when compared to their respective experimental group SS5 or SS10.

The hepatocyte growth factor (HGF) is a mitogenic protein for hepatocytes and it is the ligand of c*-*Met, which is normally expressed by cells of epithelial origin, whereas HGF is expressed in cells of mesenchymal origin. The cirrhotic animals (T0) presented a small but significant increase of HGF in serum, a further increase after 5 and 10 weeks of progress was observed, whereas the rats treated with IFC-305 showed almost a threefold increase versus control animals and only 35 to 55% versus the cirrhotic animals without treatment (Figure 6(a)). The level of HGF in the liver of cirrhotic rats (Figure 6(b)) did not present significant changes although it showed an increasing tendency in the animals treated with IFC-305. The expression of c-Met increased in cirrhotic animals at T0 (25%) with a further increase after 5 weeks of progress, followed by a decrease after 10 weeks. The treatment with the compound induced a diminution in relation with the untreated cirrhotic animals (Figure 6(c)).

### 3.7. Adenosine Receptors

**Figure 7:**
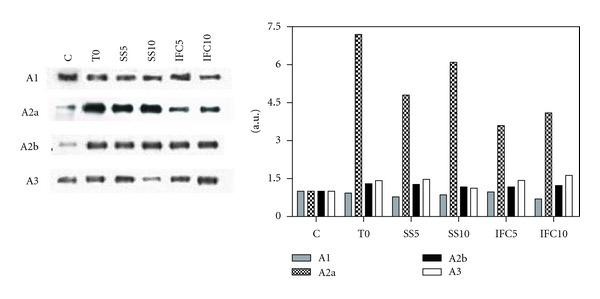
Effect of IFC305 treatment on adenosine receptors expression in CCl_4_-induced cirrhosis in rats. Expression of the indicated adenosine receptors from liver extracts was done by western blot analysis. A representative western blot image of each one is shown. The bar graph represents the densitometry analysis expressed as arbitrary units of the mean ± SEM from 3 rats/group; values were normalized to *β*-actin immunodetection (image, Figure 1(a)).

The protein expression of adenosine receptors subtypes A1, A2a, A2b, and A3 was evaluated in liver homogenate through western blot assays (Figure 7). A marked (85%) increase in A2a receptor was observed in cirrhotic animals T0 with a less increase after 5 and 10 weeks of cirrhotic progress (43 and 54%, resp.), with IFC-305 administration its expression decreased to 30%. Receptors A1, A2B, and A3 showed no significant changes in the different treatments. 

### 3.8. Mitochondrial Function and Liver ATP Content

The energy state of the liver of the experimental groups was evaluated measuring the ATP level of the hepatic tissue, and some parameters of the mitochondrial function. In Table 1, it can be observed that ATP level decreased in cirrhosis (T0), slowly recovered after 5 and 10 weeks of progress, the groups treated with IFC-305 revealed no significant decrease of ATP at 5 weeks, whereas an important elevation was observed at 10 weeks. Oxygen consumption by mitochondria, using glutamate as substrate, as well as the respiratory control and the mitochondrial potential, presented a similar profile, indicating that IFC-305 restitutes and increases energy in cirrhotic animals. 

## 4. Discussion

Regenerative function of the liver is a very complex process that has been studied after partial hepatectomy and requires a priming process before the growth stimulation factor can progress beyond the restriction points of the cellular cycle. The priming process is mediated by tumor necrosis factor (TNF*α*) and interleukin-6 (IL-6) resulting in the activation of nuclear factor *κ*B (NF*κ*B) before entering the early phase G1 [33]. The main proteins of the cell cycle are cyclins and cyclin-dependent kinases (CDKs) whose concentration is low in the quiescent state of hepatocytes, increasing in the replicative state. Their synthesis and degradation are important for cell cycle progression and are strictly regulated by the checkpoints, in order to maintain the progression of the cycle in normal conditions. However, the checkpoints are able to arrest cell cycle progression in response to DNA damage. According to these brief considerations, hepatocyte's proliferation requires a cell environment that maintains an energy balance [23], redox equilibrium, with normal synthesis and degradation of proteins, as well as mitochondrial and endoplasmic reticulum function. In cirrhosis, there is a drop in cell ATP content and mitochondrial dysfunction and a decrease in albumin synthesis [6, 24]. In the experimental cirrhosis induced by CCl_4_, there is an increase in oxidative stress generated by an increase in reactive oxygen species produced by the toxic and by mitochondrial dysfunction. Moreover, in cirrhotic rats there is a chromosomal instability highly suggestive of DNA damage and arrest of cell cycle and an increased amount of transforming growth factor *β*, a profibrogenic cytokine and a known inhibitor of liver proliferation [34]. These observations suggest that the regeneration process is a multifactorial event and that in cirrhosis several of those factors are damaged. The profound alterations of the cirrhotic liver predict an inhibition of liver proliferation. 

The present results support this possibility; Table 2 depicts a summary of the results. The cirrhotic group (T0) only showed a moderate increase of serum HGF, a marked increase in CDK6, a diminution of the cell cycle inhibitor p21 and phospho-Rb and E2F, the other proteins studied did not show changes. During the 5 and 10 weeks of cirrhosis progress, some changes occurred at 5 weeks, that is, a small increase in cyclin D1, p53, serum HGF, and its receptor c-Met; at 10 weeks of progress, a larger diminution of PCNA, cyclin B1, an increase of CDK4, p27, and serum HGF occurred with no change in the other proteins. Besides, an increase in liver ATP and mitochondrial function was observed at 10 weeks. All these changes are not sufficient to initiate the proliferative process. Clearly, there is a marked increase in cyclins, CDKs, and regulatory proteins of the cell cycle; however, only PCNA and CDK6 are overexpressed in the cirrhotic animals treated with IFC-305. An elevation in liver ATP and mitochondrial functions were found also in the latter.

The proliferating cell nuclear antigen (PCNA) is a cofactor of DNA polymerase delta and appears to be needed for both DNA synthesis and DNA repair and could be associated with cdk6/cyclin D1 [35]. This protein acts as a processivity factor encircling the DNA; thus, by creating a topological link to the genome, PCNA helps in holding DNA polymerase delta to DNA [36]. Considering the chromosomal instability characteristic in cirrhosis, it is possible that PCNA plays an important role in repairing the damaged DNA [37]. Among the cellular responses to DNA damage it is the p53-mediated activation of PCNA which binds *in vivo* to p21 and PCNA genes. p21 inhibits DNA replication but not DNA repair, the differential regulation of two DNA damage response effectors, p21/PCNA, by p53 plays an important role in DNA repair that is critical for the maintenance of genomic stability [38, 39].

As mentioned before, IFC-305 showed diverse effects during the reversion process of cirrhosis; microarray studies showed that the genes modified in cirrhosis became normalized after IFC-305 treatment. This study allowed us to visualize that the increase in PCNA and CDK6 expressions is very important to repair the DNA damage in the cirrhotic animals and to recover the genomic stability needed for the restoration of the proliferative capacity. It is possible that this is the pivotal role of action of this compound because once chromosomal instability decreased, proliferation of the liver was recovered, supported by the effect of the compound in regulating energy balance, oxidative stress, recovery of the redox state, and the capacity of protein synthesis. Controversial results of the adenosine actions could be obtained according with the different modes of action of the nucleoside that is mediated by the receptors, transporters, or through its metabolism. Then the results can depend on the experimental model used, producing contradictory results. It has been reported that extracellular adenosine and its receptors promotes fibrosis that could be blocked by the antagonist of adenosine receptors, this experiment considers endogenous extracellular adenosine in nanomolar range and uses antagonist of specific receptors and different temporality [40, 41]. In the experimental model used in this study, the recovery of cell proliferation in CCl_4_-induced cirrhosis by the adenosine derivative IFC305 was realized in a whole animal, with millimolar concentrations of adenosine in the compound, and without mitogenic stimulus. In fact, in this study we found that the overexpression of the A2a receptor protein during cirrhosis decreases in the presence of IFC-305 (Figure 7), suggesting a role of this receptor in the described effect. Previously, we showed that the inhibitory effect of IFC305 in hepatic stellate cells activation was not mediated by adenosine receptors, but it was related with adenosine transport and intracellular AMP formation [42], neither in the adenosine acceleration of the cell cycle after one-third hepatectomy [19]. Possibly, a diminution of A2a receptor expression induced by the IFC305 increases adenosine transport into the cell promoting their effects in DNA repair and maintaining the energetic equilibrium. Then, as it has been described by Fredholm and Linden, adenosine can also modulate tissue damage and repair [43, 44]. We cannot discard the participation of adenosine receptors, transporters, or the metabolic effects. Further studies are needed to elucidate the specific mechanisms involved in these processes. 

## Figures and Tables

**Table 1:** Effect of IFC305 on mitochondrial parameters in cirrhotic livers.

Treatment	(ATP) *μ*mol/gwt	State 3 Glutamate	RC Glutamate	ΔΨ ΔDO 540 nm
Controls	3.0 ± 0.7	42 ± 4	5.4 ± 0.5	191 ± 4
Cirrhosis	2.0 ± 0.16*	34 ± 3	2.8 ± 0.4*	164 ± 8*
Plus saline				
After 5 wks	2.2 ± 0.06	29 ± 3*	3.6 ± 0.3*	168 ± 10*
After 10 wks	2.8 ± 0.13	47 ± 6	4.3 ± 0.4	180 ± 6
Plus IFC305				
After 5 wks	2.9 ± 0.3**	38 ± 5	5.2 ± 0.6**	197 ± 12**
After 10 wks	3.7 ± 0.01**	48 ± 6	6.2 ± 0.5**	200 ± 4**

The results are expressed as mean ± SE of seven individual determinations. State 3 is expressed as natoms O_2_/min/mg of protein for glutamate-malate (site I) in the presence of ADP. RC: respiratory control (state 3/state 4). Statistics: ^∗^ *P* ≤ 0.01 versus the control group; ^∗∗^ *P* ≤ 0.01 versus the cirrhotic plus saline group. ATP concentration and ΔΨ were determined as described in Section 2. gwt: gram of wet tissue.

**Table 2:** Summary of the effects of IFC305 treatment on protein expression in CCl_4_-induced cirrhosis.

Protein	Cirrhosis T0	Cirrhosis progress		IFC305 treatment	
		SS5 weeks	SS10 weeks	IFC5 weeks	IFC10 weeks
PCNA	NC	↓	↓↓	↑↑↑	↑↑↑
Cyclin D1	NC	↑	↑	↑↑	↑
Cyclins E, A	NC	NC	NC	NC	NC
Cyclin B1	NC	NC	↓	↓	↓
CDK4	NC	NC	↑↑↑	↑↑↑	↑↑↑
CDK6	↑↑↑	NC	NC	↑↑↑	↑↑↑
p21	↓↓	↓↓	NC	NC	↓
p27	NC	NC	↑	NC	NC
pRb (Ser 795)	↓	NC	NC	↑	↑
E2F1	↓↓	↓	NC	↑	NC
DP1	NC	NC	NC	NC	NC
p53	NC	↑	NC	↑↑	↑↑
MDM2	NC	↓	NC	↓	NC
Serum HGF	↑	↑	↑	↑↑	↑↑
Liver HGF	NC	NC	NC	NC	NC
cMet	NC	↑	NC	↑	NC

Each group was compared to the control group. NC: no changes. ↑ or ↓: less than 50% of increase or decrease with respect to the control. ↑↑ or ↓↓: 50 to 100% of increase or decrease. ↑↑↑: more than 100% of increase.
